# Optimal dose finding for novel antimalarial combination therapy*

**DOI:** 10.1111/j.1365-3156.2012.02963.x

**Published:** 2012-04

**Authors:** S Duparc, C Lanza, D Ubben, I Borghini-Fuhrer, L Kellam

**Affiliations:** 1 Medicines for Malaria VentureGeneva, Switzerland; 2 ID-MDC Biomedical Data Sciences, GlaxoSmithKline Research and DevelopmentStockley Park West, Uxbridge, Middlesex, UK

**Keywords:** factorial design, antimalarial, drug development

## Abstract

A recent discussion meeting convened by the Medicines for Malaria Venture examined how best to manage the discovery and preclinical pipeline to achieve novel combination therapies which would address the key clinical needs in malaria. It became clear that dose optimisation of components within combination therapy was a key issue in achieving antimalarial efficacy and for preserving that efficacy against parasite resistance emergence. This paper outlines some of the specific issues in malaria that cause dose-ranging and dose-optimisation studies to be particularly challenging and discusses the potential of factorial study design to address such challenges.

## Introduction

Artemisinin revolutionised antimalarial therapy and forestalled a catastrophe. By 2001, *Plasmodium falciparum* resistance to conventional antimalarials had left many countries with no effective, affordable treatment (Roll Back Malaria 2001; World Health Organization 2010). Attendant to this was an increase in mortality, morbidity, and the frequency and severity of malaria epidemics (Roll Back Malaria 2001). The speed and extent of parasite killing with artemisinin is unprecedented. However, a short half-life requires combination with a longer-acting partner to achieve sustained cure with a 3-day treatment schedule (Alin *et al.* 1996; Giao *et al.* 2001). Today, artemisinin-based combination therapy (ACT) is highly efficacious with day-28 cure rates generally exceeding 95% (adjusted for reinfection with polymerase chain reaction genotyping), and costs less than $1 per adult treatment (World Health Organization 2010). However, ACT efficacy will not last forever; *P. falciparum* resistance is already emerging (Noedl *et al.* 2008; White 2010; World Health Organization 2011).

The spectre of untreatable *P. falciparum* rejuvenated antimalarial drug research (Roll Back Malaria 2001). It was realised that ACT was not the ‘ultimate’ antimalarial, resistance would develop, and post-artemisinin alternatives were needed. Consequently, we now have new compounds, many with novel mechanisms of action, moving from discovery into development.

Treatment of acute *P. falciparum* infection is not the only clinical objective. There are other targets that could potentially benefit from novel antimalarial combinations.

- *Plasmodium vivax* acute therapy: chloroquine resistance is an issue in some regions (Baird 2011).
- Treatment of *P. vivax* hypnozoites, the cause of clinical relapse (Galappaththy *et al.* 2007).
- Drugs that interrupt malaria transmission: a key component of malaria elimination efforts (The malERA Consultative Group on Drugs 2011).

In this revival of antimalarial drug development, safety and efficacy remain the dominant objectives. However, the need to protect new agents against the development of parasite resistance is also recognised. Thus, new antimalarials will be used in combinations with components of differing pharmacodynamics and/or pharmacokinetics. For ease of use, and to avoid their use as monotherapy, these combinations will be presented as fixed-dose formulations. Critically, doses must be optimised for use in children, whose limited immunity places them most at risk of mortality and adverse outcome (Carneiro *et al.* 2010). Formulations that increase acceptability in this target group should be a drug development priority (e.g. granules, dispersible tablets). But how do we optimize new antimalarial combinations and doses for different indications and patient populations?

## Combination and optimisation: which information?

There are no approved regulatory guidelines for antimalarial combination therapy development, but some guidance documents are relevant, such as those for fixed-dose combinations. Although experience was gained with ACT, this may not be adequate for developing the antimalarials of the future (Box 1): ACT was needed quickly, initial dose-finding was empiric and formal dose-finding studies were limited.

**Box 1 Challenges for novel antimalarial combination therapy drug development compared with the development of artemisinin-based combination therapy (ACT)**

**Table:** 

Model term	ACT	Novel combination therapies
Combination components	Artemisinin plus an existing drug used previously as monotherapy	One or more combination partners are new chemical entities
Regulatory procedure	Safety and efficacy evaluated for the combination therapy	Stringent process. Proof of concept required for monotherapy and combination therapy. Safety and efficacy evaluated for the combination therapy
Clinical data	Initial evidence mostly from the Thailand border areas in adult populations	Clinical data required from multiple centres in Asia, Africa and South America. Extensive clinical data from children required
Formulation	Loose or fixed combinations	Fixed combinations only
Paediatric formulation	Crushed adult formulation tablets used for paediatric administration. Paediatric formulations developed retrospectively in some cases. One dose fits all	Initial development of a paediatric formulation that can also be used in adults. Separate evaluation of dose and duration in children and adults
Dose finding	Empiric, based on data from monotherapy studies	Rational and optimised within combination therapy

In HIV drug development, *in vitro* activity and particularly the drug resistance profile greatly inform the pathway to choosing potential drug regimens used in clinical therapy (Zhuang & Li 2011). In tuberculosis, murine models are valuable in demonstrating the efficacy of different agents in combination (Apt & Kramnik 2009). In malaria, however, there are no validated methods that support the choice of combination partners or their relative doses (Bell 2005; Langhorne *et al.* 2011). *In vitro* studies are useful for determining drug activity by various measures, and although outcomes depend on the parasite strain and the stage mix of the parasite population, such studies are straightforward and inexpensive to perform (Noedl *et al.* 2003; Bell 2005). Drug interactions can be investigated *in vitro* using isobologram experiments (Co *et al.* 2009), though the clinical relevance of mild synergy or antagonism is not often clear (Bell 2005). Rodent models can also provide useful information, and the development and improvement of the humanised mouse model is a potentially significant advance (Arnold *et al.* 2011). However, despite an array of pre-clinical tools in malaria, when we look at these experiments with a view to designing a clinical development program there are still enormous uncertainties. Also, it is becoming clear that established tests may not be appropriate for new agents or new clinical objectives, such as transmission blocking, and new validated methods must be developed (Wein *et al.* 2010; The malERA Consultative Group on Drugs 2011).

Clinical proof-of-concept studies provide useful information on drug efficacy as monotherapy. However, these data could be misleading if the to the dose–response of the individual components is different in combination. Human pharmacokinetic studies are a regulatory requirement, and can be designed to provide useful background information to inform dose selection in combination. Such studies would need to be performed in healthy controls and patients with malaria. However, although pharmacokinetic/pharmacodynamic data can be used to justify dose choices to regulatory authorities, dose-ranging studies will provide the main evidence for the dosing rationale. Unfortunately, until the combination can be tested in malaria patients, choosing the doses for dose-ranging studies is a best estimate. We are, therefore, left with having to choose the component doses for new combinations at a late stage in clinical development.

## Factorial design: a perfect fit?

Factorial study designs offer a potential solution. A factorial design compares two or more components simultaneously. Such studies are well established in engineering applications (Myers *et al.* 2009). In drug development, they are used for animal models, but for clinical dose-finding their application has been limited mainly to hypertension (Pool *et al.* 1997). There is a precedent for using simple two-by-two factorial designs in studies of malaria combination therapy (Mulenga *et al.* 2006; Wootton *et al.* 2008). These studies compared combinations of two drugs each given at two different doses (2 × 2 matrix). Analysis was by a prospectively designed decision algorithm. However, these studies were not designed for dose-finding. To our knowledge, factorial design has not been used for dose-finding in malaria. However, it is only really with the availability of novel antimalarials and the shift to combination therapy that such a need has emerged.

Factorial study designs use a statistical model to enable interpolation of dose–response information. A quadratic model is the one most commonly used for clinical trials (Pool *et al.* 1997). This model allows for non-linearity of the dose–response. This may only be evident when the drugs are used in combination. The model is used to construct a dose–response surface that allows estimation of the optimal dose within the matrix, i.e. the model can suggest the ‘ideal’ dose even if that dose was not actually tested (see idealised example in Box 2). However, note that in clinical malaria, a placebo/placebo comparison would not be appropriate because of the risk of serious adverse outcomes. In all cases, a minimally effective dose would need to be established before designing the dosing matrix. Because the model allows data to be compared across the whole matrix rather than pairwise, sample sizes for each data collection point can be quite low. This can reduce development timelines but must be balanced against the need for safety data, so sample sizes may need to be increased to provide an adequate safety database.

**Box 2 Idealised example of a factorial study design (provided by LK)**

**Sample size grid**

Data are analysed across the whole grid, rather than pair-wise. This means that only small sample sizes are required. Note that the largest sample sizes are in the extremes of the grid (for a quadratic model).

**Table:** 

	Drug 1				
Drug 2	Placebo	Dose 1	Dose 2	Dose 3	Dose 4
Placebo	30*	10	10	10	44
Dose 1	10	10	10	10	10
Dose 2	10	10	10	10	10
Dose 3	35	10	10	10	49

* NB: A placebo/placebo arm would not be ethical in clinical malaria.

**Response**

Response data are obtained for each dose combination. In this example, high values indicate a good response.

**Table:** 

	Drug 1				
Drug 2	Placebo	Dose 1	Dose 2	Dose 3	Dose 4
Placebo	0	0.08	0.15	0.24	0.29
Dose 1	0.1	0.18	0.24	0.34	0.38
Dose 2	0.15	0.23	0.29	0.39	0.43
Dose 3	0.11	0.18	0.25	0.34	0.37

**Dose–response plot**

Based on the observed data obtained in the study, the combination of Drug 1, dose 4 and Drug 2, dose 2 would be chosen. However, as can be seen on the dose–response surface plot below, the model predicts a slightly better response, indicated by arrow at a dose that was not studied in the trial.

Note that for simplicity the data and output are presented purely as mean values. Confidence intervals are very important to show the level of precision obtained for these means and for real study output these would be presented and even drawn on the plot.

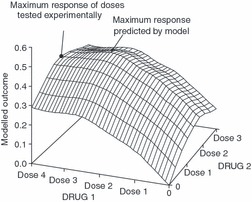

Factorial design has a number of advantages.

- Because monotherapy and combination therapy can be tested simultaneously, the contribution of each component and their interaction can be investigated in one experiment.
- The cost of a factorial study is potentially less than dose ranging on individual components followed by studies on the combination as only certain component–dose combinations are necessary to construct the dose–response surface.
- As the model includes dose regimens outside those that were actually tested, there is some flexibility should the drugs interact in an unexpected manner, for example, with significant synergy, antagonism or even both in different regions of the matrix.
- Combinations including agents with very different pharmacokinetic/pharmacodynamic profiles may be particularly suited to this design, as their interaction is difficult to predict and can be compared across a wide range of doses.
- Calculating the dose–response surface can limit unnecessary exposure to ineffective or potentially toxic drug levels. For example, as for any dose-ranging study, there is a lower dose limit at which the experiment must be interrupted to administer rescue therapy. Malaria can be deadly and patients cannot be exposed to sub-therapeutic doses for any significant time. However, factorial design offers the possibility to construct a dose–response surface in which the minimum of patients are exposed to sub-therapeutic doses. This should be at the heart of the design scheme for antimalarial combination therapies.

Balanced against these advantages, the statistical and practical input to a factorial dose-finding study is considerable. The up-front design work, including sensitivity analysis and simulations, requires specialised statistical resources. Also, managing a trial with multiple drug combinations at different doses presents practical issues. Dose choice will also be influenced by the possibility that highly effective antimalarials may have high efficacy over a large range of doses.

Another key difficulty is regarding outcomes. Antimalarials that are highly effective in rapidly reducing parasite burden may show little additional contribution in combination therapy based on clinical outcomes at day 28. For antimalarials with long half-lives, recrudescence may not be evident for longer follow-up periods. For rapidly acting blood schizontocidal antimalarials, evaluating parasite clearance rate versus an artemisinin monotherapy arm (with a subsequent long-acting rescue therapy) may be possible, but requires clinical validation. For combinations of antimalarials with diverse pharmacodynamic/pharmacokinetic profiles, it may be necessary to evaluate more than one outcome. This further complicates statistical modelling for factorial designs. In vulnerable patients, such as children, it is critically important to have information on the minimally effective doses before including such patients in trials that can then be designed to optimise dose–response above this threshold. Consequently, further data may need to be collected from studies in adults with malaria, or by using alternative endpoints, such as human challenge experiments or clearance of asymptomatic parasitaemia before proceeding.

The availability of novel antimalarial candidates provides an opportunity to test new approaches to dose optimisation for combination therapy, such as factorial design. Defining appropriate outcomes to adequately differentiate between drug and dose combinations targeted at specific clinical objectives will be a key consideration. However, as a pioneering methodology in antimalarial drug development, factorial design should be considered on a case-by-case basis as a potential tool to optimise the efficacy, safety and longevity of future antimalarial combination therapies.
